# A Synergistic Effect of Reactive Oxygen and Reactive Nitrogen Species in Plasma Activated Liquid Media Triggers Astrocyte Wound Healing

**DOI:** 10.3390/ijms21093343

**Published:** 2020-05-08

**Authors:** Eloisa Sardella, Maria Grazia Mola, Roberto Gristina, Monica Piccione, Valeria Veronico, Manuela De Bellis, Antonio Cibelli, Maura Buttiglione, Vincenza Armenise, Pietro Favia, Grazia Paola Nicchia

**Affiliations:** 1 Institute of Nanotechnology, National Research Council of Italy (CNR-NANOTEC), c/o Department of Chemistry, University of Bari Aldo Moro, 70126 Bari, Italy; eloisa.sardella@cnr.it (E.S.); roberto.gristina@cnr.it (R.G.); 2 Department of Biosciences, Biotechnologies and Biopharmaceutics, University of Bari Aldo Moro, 70125 Bari, Italy; mariagrazia.mola@uniba.it (M.G.M.); m.piccione2991@gmail.com (M.P.); manuela.debellis84@gmail.com (M.D.B.); antonio.cibelli@einstein.yu.edu (A.C.); 3 Department of Chemistry, University of Bari Aldo Moro, 70126 Bari, Italy; valeriaveronico@gmail.com (V.V.); vincenza.armenise@uniba.it (V.A.); 4 Department of Biomedical Sciences and Human Oncology, Medical School, University of Bari Aldo Moro, 70124 Bari, Italy; maura.buttiglione@uniba.it

**Keywords:** astrocytes, wound healing, plasma activated medium, oxidative stress

## Abstract

Astrocyte proliferation and migration toward injured Central Nervous System (CNS) areas are key features of astrogliosis and glial scar formation. Even though it is known that intracellular and environmental Reactive Oxygen and Nitrogen Species (RONS) affect astrocyte behaviour in physiological and pathophysiological conditions, their effects on the migration and growth of astrocytes are still unclear. Plasma-technologies are emerging in medicine as a tool to generate RONS for treating cells directly or through Plasma Activated Liquid Media (PALM). In this paper, we show for the first time how the use of PALM can modulate both astrocyte growth and migration as a function of active species produced by plasma in liquids. Our results show that PALM, generated by means of cold atmospheric pressure plasmas fed with N_2,_ air or O_2_, can modulate astrocyte behaviour depending on the content of hydrogen peroxide and nitrite in the liquid. In particular, H_2_O_2_ enriched PALM induced a negative effect on cell growth associated with the mild wound healing improvement of primary astrocytes, in a scratch assay. Nitrite enriched PALM induced a selective effect on the wound healing without affecting cell growth. PALM containing a more balanced level of H_2_O_2_ and NO_2_^−^ were able to affect cell growth, as well as significantly ameliorate wound healing. None of the PALM investigated induced upregulation of the gliotic inflammatory marker glial fibrillary acidic protein (GFAP), or of the astrocyte markers Aquaporin-4 (AQP4) and Connexin-43 (Cx-43) analysed by Western blot. Finally, immunofluorescence analysis revealed the presence of NO_2_^-^ able to induce elongated protrusions at the front end of wounded astrocytes in the direction of cell migration. With our study we believe to have shown that PALM offer a novel tool to modulate astrocyte behaviour and that they are promising candidates for controlling astrogliosis in the case of CNS injuries.

## 1. Introduction

Astrocytes are the central component of the so called “neuro-glio-vascular unit” [1,2,3], playing several homeostatic functions and a central neuroprotective role in the Central Nervous System (CNS). In response to a wound caused by a wide range of neurological insults such as trauma, stroke, infections, tumours, neurodegenerative diseases and epilepsy, astrocytes become “reactive” and undergo astrogliosis, a neuro repair inflammatory process characterised by their proliferation and migration toward the site of injury. As in other organs and tissues, astrocyte reaction is a protective mechanism aimed at restoring CNS homeostasis and at healing the wound. However, depending on its intensity and duration, astrogliosis can become detrimental when reactive astrocytes form a “glial scar” that inhibits the axonal regeneration and the recovery of function [4,5]. The upregulation of the cytoskeleton GFAP is widely considered the marker for astrogliosis [6], as well as the alteration of key astrocyte channel proteins, such as AQP4 and Cx43 [7,8,9].

During astrogliosis, as well as in conditions of ischemia and stroke-induced hypoxia and during neurodegenerative processes, astrocytes face oxidative/nitrosative stress due to the release and accumulation of Reactive Oxygen and Nitrogen Species (RONS) [10] above the level normally controlled by antioxidant enzymes and molecules. Chronic astrogliosis can result in a persistent production of RONS with a consequent general neurotoxic effect. However, in accordance with their important neuroprotective role, under oxidative/nitrosative stress astrocytes exert important redox-scavenger properties [11]; the failure of their function, whether or not induced by RONS, has been directly associated with several CNS disorders [12,13,14,15,16,17,18]. Therefore, modulating astrocyte function could delay neurodegenerative processes, such as Parkinson’s and Alzheimer’s diseases, in which, as an example, H_2_O_2_ is a paramount molecule causing neuronal loss. 

An innovative and controlled way to produce RONS is represented by plasma processes, with setups properly designed for medical and clinical purposes [19,20]. The acronym CAP, Cold Atmospheric Plasmas, is often used to define non equilibrium plasmas at atmospheric pressure. Cold plasmas are ionised gases including radicals, neutral and charged particles, excited species and vis-UV radiations, close to room temperature, in a non-thermodynamic equilibrium. Plasma Jets and Dielectric Barrier Discharges (DBD) [21] are the most utilised CAP sources in Plasma Medicine, some of them licensed for clinical applications and available on the market for uses in oncology and wound healing [22,23]. Due to the low temperature, the use of CAP for medical applications is well assessed for sterilisation and for the surface modifications of biomedical materials [21,24,25]. 

Primary RONS (e.g., NO, NO_2_, O_3_, OH radicals) are produced from the feed gas in the discharge. Once generated, they can diffuse into the biological target (i.e., tissue, cells) and/or into the water-based liquid surrounding the biological target (direct treatments). When a liquid of biological interest like water, saline solution or cell culture media faces the plasma without the presence of the biological target, primary RONS in the plasma phase form secondary RONS (e.g., ●OH, ●NO, ONOO^−^, H_2_O_2_, NO_2_^−^ and NO_3_^−^ ions) through different chemical pathways. Such liquids can be applied in contact with the biological target (indirect treatments) or systemically administered to the patient, and they could potentially be used for therapeutic purposes (i.e., blood coagulation, anti-cancer effects, cell proliferation) [26,27]. These liquids activated by plasma can be called Plasma Activated Liquid Media (PALM).

The nature and density of the secondary RONS depend on the chemical composition of the exposed liquid as well as on the experimental parameters of the particular ignited CAP. The confinement of the plasma source in a sealed system, with limited external air contamination, allows for better control of the nature and dose of the RONS. PALM are utilised as vectors for delivering CAP-generated RONS for skin wound healing and to induce cancer cell death [28,29,30]. However, despite many studies concerning the direct and indirect exposure of eukaryotic cells to plasmas, controversies still exist on how to rationalise the mechanism of interaction between plasma-generated RONS and cells. The hypothesis of our study is that PALM can be used as a controllable way to modulate typical astrocyte behaviour associated with their neuroprotective role in wound healing and to identify key players of astrocytic oxidative/nitroxidative stress. Due to the reactivity of most of the species produced by the gas feed and their short lifetime, PALM contain essentially stable species like H_2_O_2_, NO_2_^−^ and NO_3_^−^. Even though PALM are more than nitrite and hydrogen peroxide solutions [31], we focused our attention on NO_2_^−^, which is proposed to have potential therapeutic effects, and on H_2_O_2_, able to act as a mediator of adaptive responses in astrocytes [32]. Moreover, nitrite and hydrogen peroxide are final products of complex reactions involving other RONS species. 

PALM with different compositions in terms of the H_2_O_2_ and NO_2_^−^ content have been produced and used to ascertain their potential effects on astrocyte growth, migration and GFAP, AQP4 and Cx43 expression levels. We here demonstrate that astrocytes are sensitive to PALM with a reaction that tightly depends on the relative concentration of H_2_O_2_ and NO_2_^−^ ions in the PALM.

## 2. Results

### 2.1. Different PALM Compositions as a Function of the Gas Feed 

**Figure 1:**
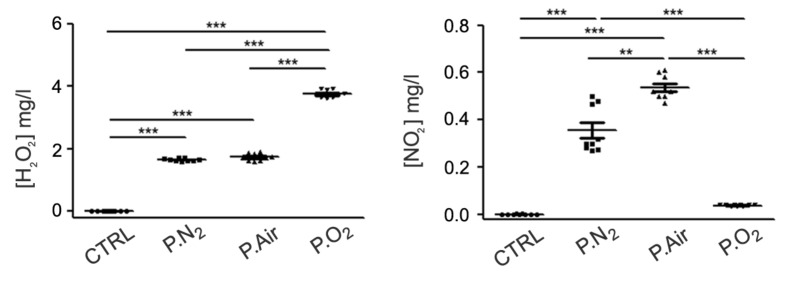
Chemical composition of the different Plasma Activated Liquid Media (PALM). Concentration of H_2_O_2_ and NO_2_^−^ in untreated medium (Ctrl), in PALM N_2_ (P.N_2_), in PALM Air (P.Air) and in PALM O_2_ (P.O_2_). The following experimental conditions were kept constant: 6 KHz, 13 kV, 100 ms period, 25% D.C; 30 s treatment time, 0.5 slm gas feed. Significant differences of the means were calculated by One-way Anova followed by the Newman–Keuls Multiple Comparison Test. *: *p* < 0.05; **: *p* < 0.01; ***: *p* < 0.001.

In order to investigate how the gas feed affects the chemical composition of the PALM, different PALM were produced with N_2_ (P.N_2_), synthetic air (P.Air) or O_2_ (P.O_2_). As shown in Figure 1, the concentration of H_2_O_2_ produced in the different PALM was high in P.O_2_ but moderate in P.Air and in P.N_2_. Despite the absence of O_2_ in the gas feed, H_2_O_2_ was detected in P.N_2_, probably due to the involvement of water vapor in the plasma process, through precursors such as atomic oxygen (O), hydroxyl radical (•OH), superoxide anion (O_2_•^−^) and singlet oxygen (^1^O_2_) [33]. When P.N_2_ was used, the amount of NO_2_^−^ in the corresponding PALM increased, even though its highest concentration was observed in P.Air. 

These results demonstrate the active role of O_2_ in the production of nitrites in the PALM. In fact, in P.N_2_ water vapor was the only source of oxygen, while in the case of synthetic air the simultaneous presence of water vapor and O_2_ from the feed gas increased the production of nitrites. Due to the absence of N_2_, the amount of nitrite ions found in P.O_2_ was comparable to that found in the control medium. These results show that P.N_2_-treated PALM were characterised by a moderate level of ROS and RNS (RONS PALM), P.Air by a moderate level of ROS and a high level of RNS (RNS PALM), and P.O_2_ by a low level of RNS and a high level of ROS (ROS PALM). These three PALM were then used to test the effect of their relative RONS content on primary cultured astrocyte behaviour in the following assays.

### 2.2. Effect of PALM Containing Different Ratios of ROS and RNS on Primary Astrocyte Cultures

**Figure 2:**
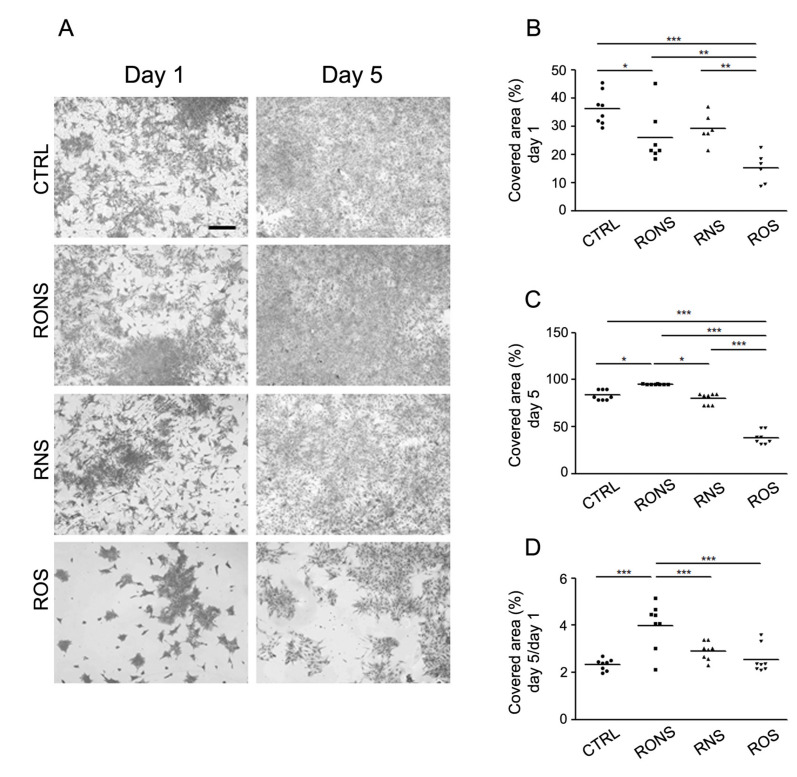
Coomassie blue assay of astrocytes incubated with different PALM. (**A**) Coomassie blue staining of astrocytes, one and five days after a 2 h-PALM incubation. Images are compared with the untreated medium (Ctrl) used as control. Different PALM are indicated as follows: PALM N_2_ (RONS), PALM Air (RNS) and PALM O_2_ (ROS). Scale bar, 500 μm. (**B**) Scattered plots showing the percentage area covered by cells at day 1. (**C**) Scattered plots showing the percentage area covered by cells at day 5. (**D**) Scattered plot measuring the ratio of the area covered by astrocytes after five days over that covered after one day. A One-way Anova and Newman–Keuls Multiple Comparison Test were performed. *: *p* < 0.05; **: *p* < 0.01; ***: *p* < 0.001.

The first set of experiments was aimed at analysing the astrocyte growth, after one and five days following 2 h of PALM incubation (Figure 2). By looking at the substrate area covered by cells, the effect of PALM incubation after one day (Figure 2A,B) resulted in a mild decrease of the area covered by cells induced by PALM containing RONS and ROS, which was greater in the presence of high levels of ROS (ROS PALM). In contrast, the results using the different PALM were different when the cells were analysed after five days (Figure 2A,C). In particular, RONS PALM showed a covered area similar to the one shown for control cells, while ROS PALM confirmed their effect on astrocyte growth, which did not seem to be affected by RNS PALM at day 5 or at day 1. More details were provided by the analysis of cell growth, shown as the ratio of the area covered by the cells at five days over the area covered at one day. As shown in Figure 2D, the growth effect of RONS PALM on astrocyte growth was more prominent during this time window. 

This information better highlights that a balanced level of ROS and RNS induces a negative effect on astrocyte growth during the first 24 h and is able to produce a positive effect thereafter. In contrast, ROS PALM did not affect astrocyte growth from day 1 to day 5, indicating that the major effect of ROS-enriched PALM (ROS PALM) seemed limited to the first day. 

### 2.3. ROS and RNS Are Able to Improve Astrocyte Migration without Inducing a Gliotic Reaction

**Figure 3:**
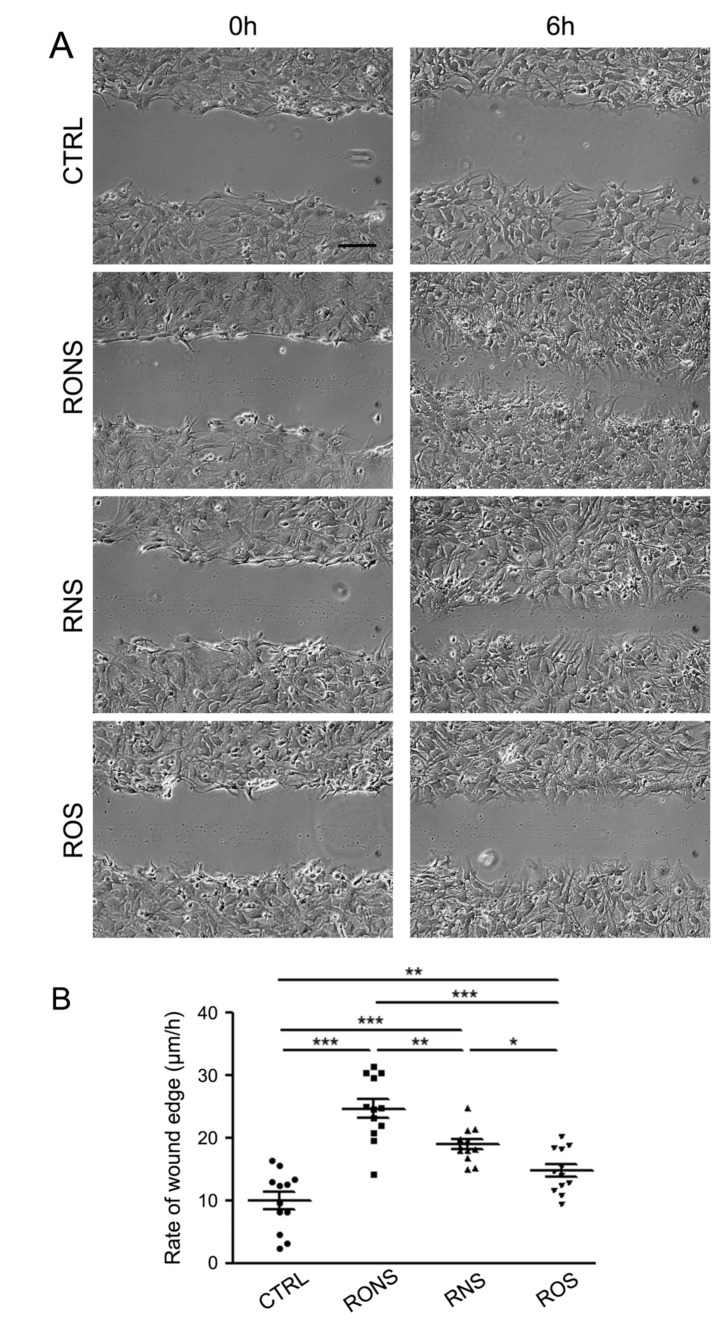
Scratch-induced migration assay in PALM-incubated astrocyte monolayers. (**A**) Phase-contrast micrographs showing astrocytes exposed to untreated medium (Ctrl) and to P.N_2_ (RONS), P.Air (RNS) and P.O_2_ (ROS) PALM immediately after the scratch (0 h), and 6 h post-wounding. Scale bar, 100 μm. (**B**) Scattered plots showing the mean ± SE values of the migration rate of astrocytes incubated as indicated. A One-way Anova and Newman-Keuls Multiple Comparison Test were performed: *: *p* < 0.05; **: *p* < 0.01; ***: *p* < 0.001.

The effect of the three PALM was tested on the migration rate of astrocytes by means of the scratch-induced wound healing assay (Figure 3). 

The assay was performed in the presence of a reduced amount of serum in the medium to minimise the effect of cell proliferation. The rate of astrocyte migration, measured 6 h after the initial scratches, was significantly improved with all PALM, independently of their composition. The highest wound closure rates were measured in cells exposed to RONS PALM, followed by cells exposed to RNS PALM, and then to those exposed to ROS PALM (Figure 3A,B).

**Figure 4:**
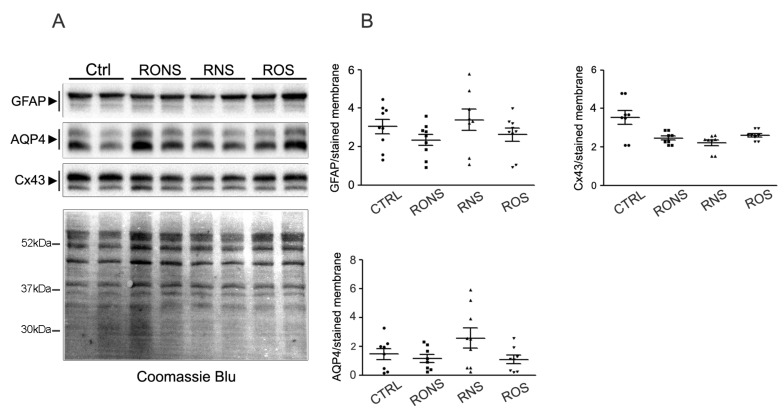
GFAP, AQP4 and Cx43 immunoblotting and quantification in PALM-incubated astrocytes. (**A**) Representative Western blot analysis of GFAP, AQP4 and Cx43 expression in astroglial cultures exposed to untreated medium (Ctrl), to P.Air (RNS), to P.O_2_ (ROS) and to P.N_2_ (RONS). Protein samples were collected 24 h after 2 h-PALM incubation. (**B**) Summary of the densitometric analysis of GFAP, AQP4 and Cx43 corresponding signals normalised to the Coomassie blue-stained membrane. No significant differences by One-way Anova test were found between PALM-treated and Ctrl astrocytes.

To assess whether the PALM-induced increase in astrocyte migration was associated with cell activation, the astrogliotic marker GFAP was monitored in the cells after incubation with the three different PALM. The potential alteration of key protein channels whose alteration has been associated with astrogliosis, such as the water channel AQP4 and the gap-junctional protein Cx43, was also analysed in parallel (Figure 4). The expression levels of GFAP, AQP4 and Cx43 were analysed after 24 h of growth by means of a Western blot analysis, (Figure 4A) and quantified by densitometry (Figure 4B). 

**Figure 5:**
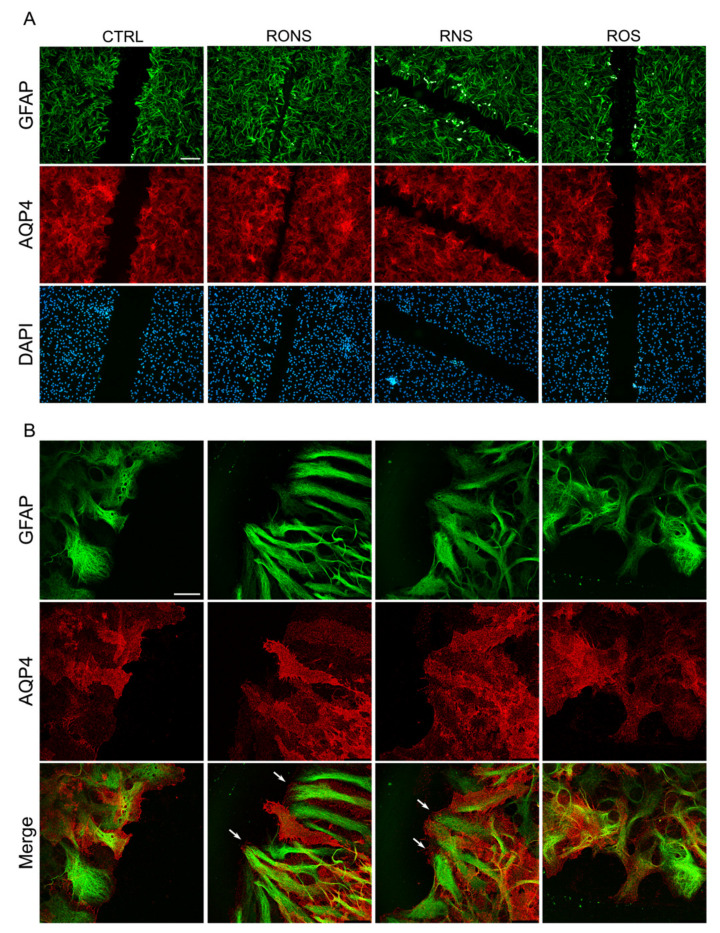
GFAP and AQP4 protein localisation in astrocyte cultures after PALM incubation. Immunostaining at 6 h after scratch for AQP4 (red) and GFAP (green) in PALM-exposed and control astrocytes. Untreated medium (Ctrl), P.N_2_ (RONS), P.Air (RNS) and P.O_2_ (ROS) PALM. (**A**) Fluorescence signal for AQP4 and GFAP in migrating cells at the wound. Nuclear staining with DAPI is also shown. (Scale bar: 100 μm). (**B**) Confocal images at the leading edge of wounded astrocytes as in (**A**). Merged images (Merge) of the two proteins’ localisation are reported (Scale bar: 25 μm). White arrows in RONS and RNS indicate enhanced foot processes at the front end of the scratch.

The results obtained show that the expression levels of GFAP, AQP4 and Cx43 proteins remained unaltered in rat astrocytes exposed to different PALM compared with untreated control astrocytes. The GFAP and AQP4 expression and localisation were also evaluated in PALM-treated astrocytes by performing immunofluorescence (Figure 5A) and confocal imaging (Figure 5B).

In line with immunoblotting data, both immunostained proteins remained unchanged in terms of the expression level and distribution pattern. These findings suggest that the PALM treatment performed on astrocytes, at the doses applied in our experimental conditions, while affecting their migration rate in the wound healing test did not seem to promote a gliotic reaction in our in vitro conditions. Interestingly, the confocal analysis of the wound leading edge indicates that the migration of astrocytes exposed to RONS PALM and to RNS PALM is associated with enhanced plasma membrane foot processes at the front end of the scratch, compared to control astrocytes (Figure 5B and Figure S1). These effects were not revealed in astrocytes exposed to ROS PALM. Such results indicate that the morphological changes in the form of enhanced foot processes at the front end of the scratch are strictly dependent on the synergistic effect of nitrites and hydrogen peroxide and, in particular, on the presence of RNS in the PALM.

## 3. Discussion

Despite numerous studies investigating how the pathological content of RONS contributes to neurodegenerative diseases and aging, the relationship between RONS and cell behaviour under normal physiology, and in diseased CNS, remains largely unclear. In this study, we have investigated whether astrocytes are influenced by PALM-derived RONS and, in particular, whether they are able to trigger key mechanisms of astrocyte activation and gliotic reaction such as cell growth, migration and alteration of gliotic and astrocyte markers. 

The approach proposed in our paper is based on an indirect exposure of cells to plasma effects mediated by PALM. In PALM, the effect of short-lived species, such as ONOO^−^, can be considered negligible compared with other stable species, due to preferential reactions with medium components at physiological pH (~6.8) [34]. It is worth mentioning that, although other RONS species are present in PALM, those investigated in our paper, H_2_O_2_ and NO_2_^−^, can be considered, with good approximation, as markers of all ROS and RNS species produced in PALM, respectively, due to their stability [27].

The main finding of our study is that extracellular RONS delivered to astrocytes after 2 h exposure to P.N_2_, P.Air or P.O_2,_ are indeed able to trigger key responses associated with the neuroprotective role of astrocytes. Different combinations of RONS-containing PALM can modulate proliferation and promote migration without inducing a gliotic inflammatory reaction. Interestingly, these effects might be associated with marked changes in the glial protrusion morphology at the front edge of scratches, indicating that increased migration involves an alteration in the cell volume and cell cytoskeleton as occurs in vivo [35].

ROS species above a certain concentration seem to have effects on cell growth, which is consistent with the appearance of an oxidative stress condition. In the absence of RNS, they can exert a mild positive effect on cell migration and wound healing not associated with changes in cell morphology. The role of ROS in accelerating astrocyte migration could be dependent on the activation of ERK1/2 and JNK1/2 cascades, as shown by Hsieh et al. [36], but does not seem to be related to changes in the cell shape or cell cytoskeleton.

In combination with very low concentrations of H_2_O_2_, the presence of RNS species can affect astrocyte growth and exert a mild growth effect depending on the combination with ROS. RNS species always play a more important role in ameliorating wound healing, and their effect on astrocyte migration is associated with changes in astrocyte morphology, indicating the potential activation of a different signalling cascade involving the cell cytoskeleton. 

Despite several papers attesting the driving force of H_2_O_2_ in producing desired responses [37,38], and others attesting that exogenous RNS species produced in PALM are also implicated in neuronal differentiation, proliferation and axonal growth [39], the clear importance of determining the correct combination of RNS and ROS to promote the desired cell responses is reported here for the first time. These results demonstrate that a synergistic effect of ROS and RNS (i.e., H_2_O_2_ and NO_2_^−^) can trigger specific RONS-based cell functions in astrocytes, as already shown for tumour cells [32]. 

From a physiological point of view, it is well known that NO_2_^-^ ions are an important reservoir of nitric oxide (NO) in the vascular system [40]. Spectroscopic investigations show the occurrence of NO in discharges in air or in N_2_, and the presence of nitrite and nitrous acid in plasma-treated water [41]. In non-buffered acid media, the disproportionation of HNO_2_ increases the concentration of nitrates and the formation of NO, to which the therapeutic role is attributed [41]. The presence of phosphate buffer in DMEM 10% FBS used in this study stabilises the cell medium at a pH ranging from 6.8 to 7.4, so this reaction does not occur. However, an active role of NO in the observed astrocyte behaviour cannot be excluded. NO is produced by all brain cells, including neurons, endothelial cells and glial cells (astrocytes, oligodendrocytes and microglia), by Ca^2+^/calmodulin-dependent NO synthase (NOS) isoforms, for the control of cerebral blood flow and neurotransmission [42]. Moreover, the involvement of NO in neurodegenerative diseases opens the possibility of pharmacological treatments directed at NO metabolic pathways. The future application of plasma-activated liquid media enriched by NO precursors like NO_2_^−^ can thus be envisioned in this context. 

The gliotic reaction, like any inflammatory reaction outside the CNS, is aimed at repairing the damage and is therefore beneficial in its scope. As the isolation of the insulted area is the absolute priority of an inflammatory reaction, if exacerbated it can produce detrimental effects mainly dealing with scar formation and the subsequent loss of tissue function. This effect is usually transient in tissues capable of regenerating (for example the epidermis), while it can be permanent in the CNS, the regenerating properties of which are highly limited, with major consequences related to the loss of function, depending on the specific area involved. Our results suggest that tuning the relative concentrations of NO_2_^−^ and H_2_O_2_ allows for the modulation of astrocyte proliferation and migration, avoiding a gliotic reaction. Moreover, the PALM conditions used in this study produce important effects on astrocyte responses without altering the expression levels of the gap junctional protein Cx43 and the water channel protein AQP4, indicating that the oxidative stress induced is likely to be moderate, with no effect on astrocyte homeostatic function. The absence of a PALM-induced inflammatory reaction (unaltered GFAP) or of a PALM-induced alteration of cell homeostatic functions (unaltered AQP4 and Cx43) is an important finding of the present study, in view of the potential therapeutic use of PALM. In addition, having the possibility of using PALM to modulate the key behaviour of the astrocyte reaction, such as growth and migration, can provide important information and molecular cues useful for a better understanding of the neuroprotective role of astrocytes. 

In our experiments, we are probably below the toxicity threshold of both H_2_O_2_ and NO_2_^−^, so this probably allows for the promotion of positive effects on astrocytes through PALM. In the literature [43], it is reported that the use of rotenone, a broad-spectrum pesticide able to promote the formation of RONS, induces the astrocyte production of NO_2_^−^ in the hippocampus at concentrations around 35 μM, even higher than the values found in our research (~6 μM). Our results show that while NO_2_^−^ promotes both proliferation and migration, in the case of H_2_O_2_ we have an inhibition of astrocyte growth and a mild increase in astrocyte migration compared to the control. Further studies are necessary to investigate the molecular mechanisms underpinning this different effect, which is likely to be linked to a different effect on signalling pathways involved in astrocyte growth. We can state that the results here shown indicate a different effect of NO_2_^-^ and H_2_O_2_ in the signalling pathway controlling astrocyte growth and migration. However, as mentioned before, due to the complexity of the chemical composition of the cell culture medium before and, even more so, after plasma irradiation, it is reasonable to assume that other molecules can contribute to such an effect [44]. 

In conclusion, the PALM produced in this study were found to be suitable to promote the interaction between RONS and astrocyte responses. We believe this study provides a novel tool for future investigations aimed at detailing the switch line between the beneficial and harmful effects of oxidative stress on astrocytes, also allowing a better understanding of aging as well as Alzheimer’s and Parkinson’s diseases, ALS and stroke [4], in which oxidative stress plays a key role.

## 4. Materials and Methods

### 4.1. Ethics Statement

In this study, no experiments were performed on live animals. Primary cell cultures were prepared from Wistar rat pups according to the European directive on animal use for research and the Italian law on animal care. The protocol was approved on 27 September 2017 by the Italian Ministry of Health (Protocol no. 710/2017-PR). Wistar rats (4 months old), originally from Charles River, were bred in the Animal Facility of the Department of Biosciences, Biotechnologies and Biopharmaceutics, and the pups were used for brain explant and cell culture preparation. Rats were maintained under a 12-h dark-to-light cycle, at a constant room temperature and humidity with food and water ad libitum in 1290D Eurostandard Type III cages (Tecniplast, Varese, Italy).

### 4.2. Primary Astrocyte Cultures

Primary cultures of Wistar rat cortical astrocytes were prepared from P0-P1 brains of pups sacrificed by cervical dislocation. Normally, eight brains from the entire littermate were pulled for each preparation. Cells were cultured in DMEM (Cat: ECM0103L; Euroclone, Milan, Italy) with stable L-glutamine (Cat: ECB3000D; Euroclone, Milan, Italy) supplemented with 10% fetal bovine serum (FBS) (Cat: ECS0180L; Euroclone, Milan, Italy), 100 U/mL penicillin and 100 mg/mL streptomycin (Cat: ECB3001D; Euroclone, Milan, Italy), and maintained in a humidified incubator with 5% CO_2_ for 3–5 weeks, as described in Mola et al. [45]. Microglial and progenitor cells were detached by shaking before each change of medium for the first two weeks. Astrocyte purity was determined by GFAP staining. More than 95% of the cells were type-1 cortical astrocytes. All cell culture products were purchased from Euroclone (https://www.euroclonegroup.it).

### 4.3. Plasma Source and Plasma Parameters Utilised for the Synthesis of PALM

The “modified PetriPlas+” plasma source utilised in this research was a volume DBD able to treat, in remote, 3 mL of liquid contained in commercial Petri dishes (57 mm dia. TPP, GER). The distance between the plasma and the surface of the liquid was set at 3 mm. The source consisted of a stainless steel ground grid 4 mm away from a high voltage electrode made of a quartz-covered copper disk (30 mm dia.). Two silicon gaskets allowed the Petri dish to be fitted to the small volume between the discharge and the flow unit and sealed. A dry diaphragm pump (Pfeiffer) was used to keep the working pressure constant (750 mTorr), as measured with a MKS baratron. Experiments were run at room temperature, with a flow rate of 0.5 l min^−1^ of synthetic air (Air Liquide, 99.999%), O_2_ (Air Liquide, 99.999%) or N_2_ (Air Liquide, 99.999%). The gas flow rates were set using MKS mass flow controllers. Before any treatment, the source was purged for 1 min with the feed gas. The discharges were driven with an alternating-current (AC) power supply connected to a TG1010A programmable 10 MHz DDS TTi function generator; the electrical parameters were monitored with a Tektronix TDS 2014 C digital oscilloscope and a Tektronix P6915A HV probe. The applied voltage was set to 13 kV, and the frequency was kept constant at 6 kHz. The charge Q transported in the discharge was determined indirectly through a 100 nF capacitor. The discharges run for this research were pulsed with a 25% duty cycle (D.C.), 25 ms of plasma on (t_on_) over a period (t_on_ + t_off_) of 100 ms. The dissipated energy was calculated by multiplying the mean energy per cycle by t_on_. Treatments of 30 s were performed. Fresh PALM were used within 15 min after activation for chemical analysis and for biological experiments. The plasma conditions investigated are reported in Table 1, along with the measured dissipated energy.

### 4.4. H_2_O_2_ and NO_2_^−^ Detection

A Spectroquant® Hydrogen Peroxide Test (Cat: 118789; Merckmillipore, Burlington, MA, USA; http://www.merckmillipore.com) was used for the detection of H_2_O_2_ in PALM. In the presence of a phenanthroline derivative, H_2_O_2_ reduces Cu^2+^ to Cu^+^ ions, which form an orange-colored phenanthroline complex that is determined photometrically at 445 nm. The samples were analysed within 15 min after the plasma treatment. Nitrites ions were detected by means of the Griess assay (Spectroquant test kit, Cat: 114776; Merckmillipore, Burlington, MA, USA); nitrite ions in acid solution react with sulfanilic acid to form a diazonium salt, which in turn reacts with N-(1-naphthyl)-ethylenediaminedihydrochloride to form a red–violet azo-dye determined at 525 nm. The samples were analysed within 15 min after the plasma treatment. 

### 4.5. PALM Treatment of Primary Astrocyte Cultures

4 × 10^4^ astrocytes were seeded 40 h before PALM incubation. PALM were synthesised from 3 mL of medium and immediately kept in contact with astrocytes. After 2 h of incubation at 37 °C, the PALM were removed and replaced with fresh untreated DMEM. Astrocytes treated with different PALM were analysed for growth, migration and protein expression in blind testing by at least three different researchers, independently. We performed each set of experiments using three different astrocyte preparations. 

### 4.6. Coomassie Blue Cell Staining and Surface Area Covered by Cell Calculation 

The behaviour, in terms of both growth and morphology, of astrocytes grown on 24 multiwell-plates, for one and five days after PALM incubation, was investigated by means of the Coomassie Blue assay. In brief, cells were fixed with 4% paraformaldehyde in PBS for 20 min, then exposed to a staining solution of 0.2% Coomassie Blue solution (Coomassie Brilliant Blue R250, Biorad, Hercules, CA, USA; http://www.bio-rad.com) in 10% Acetic Acid, 45% methanol and 45% H_2_O) for 3 min. Stained cells were observed using a Nikon Eclipse Ti inverted optical microscope. At least seven images/condition were acquired with a Nikon DS Fi2 CCD camera. An Image J analysis was performed to calculate the percentage of substrate area covered by cells. Scatter plots were built together with the mean ± S.E.M (Standard Error of the Mean) of the substrate area covered by cells. 

### 4.7. Scratch-Induced Migration Assay

The migration assay was performed on astrocytes grown on poly-L-lysine-(PLL)-coated glass bottom dishes, 20 mm diameter (Cat: 627860; Greiner-Bio-One, Kremsmünster, Austria; https://www.gbo.com). After PALM incubation, astrocytes were incubated with fresh DMEM containing 1% FBS to reduce the effect of cell proliferation. In each dish, four areas of the monolayer were scratched with a sterile 200 μL pipette tip, creating four gaps of similar widths of approximately 200 μm. The scratch-wounded monolayers were rinsed twice with the medium to remove detached cells. Phase-contrast light micrographs were taken immediately (time zero) after the scratch and 6 h later using a digital camera connected to a Nikon microscope (10× objective). The widths of the scratches were measured with ImageJ software, and the wound healing rate was measured through the following formula:

gap closure rate (*micron/hour*) = (gap_t6_ – gap_t0_) (*micron*)/6 (*hours*)

where gap_t6_ and gap_t0_ are the distances between the borders of the scratch after 6 h and at time zero, respectively. Data for each experimental condition are collected from seven to ten different measurements of three sets of independent experiments performed on different cell preparations. For each set of experiments at least two different dishes were analysed.

### 4.8. SDS-PAGE and Western Blot Analysis

Protein samples of astrocytes grown on PLL-coated glass bottom dishes, 20 mm diameter (Cat: 627860; Greiner Bio-One, Kremsmünster, Austria), were prepared 24 h after PALM treatment, as previously described [46]. In brief, cells were rinsed with ice-cold PBS and scraped in RIPA buffer (25 mM Tris–HCl, pH 7.6; 150 mM NaCl; 1% Triton X-100; 1% sodium deoxycholate; 0.1% SDS) added with a proteinase inhibitor cocktail (Cat: 11697498001; Roche Life Science, Penzberg, Germany; https://lifescience.roche.com), sonicated for 10 s on ice and centrifuged at 21,000× *g* for 45 min at 4 °C. The protein concentration in the supernatant was quantified using a bicinchoninic acid (BCA) Protein Assay Kit (Cat: 23225; Thermo Fisher Scientific, Waltham, MA, USA,; http://www.thermoscientific.com). 18 μg protein/lane was separated on 13% SDS-PAGE and transferred to polyvinylidene fluoride membranes (Cat: IPVH00010; Merckmillipore, Burlington, MA, USA). The membranes were probed for 2 h with primary antibodies, then rinsed and incubated with the appropriate peroxidase-conjugated secondary antibodies. Immunoreactive bands were revealed using an enhanced chemiluminescence detection system (ECL Substrate, Bio-Rad, Hercules, CA, USA) and visualised on a Chemidoc imaging system (Bio-Rad, Hercules, CA, USA). The signal was quantified with Image Lab software 5.2.1. The density values of target bands were normalised to the total stained proteins for each lane profile. 

### 4.9. Antibodies

The following primary antibodies were used: rabbit anti-AQP4 (Cat: sc-20812; Santa Cruz Biotechnology, Dallas, TX, USA; https://www.scbt.com) dilution 1:500, rabbit anti-Cx43 (Cat: C6219; Sigma, Saint Louis, MI, USA; https://www.sigmaaldrich.com,) dilution 1:2000 and mouse anti-GFAP (Cat: G3893; Sigma, Saint Louis, MI, USA) dilution 1:500. The following secondary antibodies were used: AlexaFluor 488-conjugated donkey anti-mouse IgG (Cat: A21202) and AlexaFluor 594-conjugated donkey anti-rabbit IgG (Cat: A21207) (all from Thermo Fisher Scientific, Waltham, MA, USA) for immunofluorescence at a dilution of 1:1000; peroxidase-conjugated goat anti-rabbit IgG (sc-2004) and goat anti-mouse IgG (sc-2005) (all from Santa Cruz Biotechnology, Dallas, TX, USA) for Western blot at a dilution of 1:5000. 

### 4.10. Immunofluorescence and Confocal Microscopy Analysis

Astrocytes grown on 20 mm diameter PLL-coated glass bottom dishes (Cat: 627860; Greiner Bio-One, Kremsmünster, Austria) were fixed in 4% paraformaldehyde, rinsed in PBS and permeabilised for 20 min with 0.3% Triton X-100 (Cat: T8787; Sigma, Saint Louis, MI, USA) in PBS (Euroclone, Milan, Italy). After blocking with 0.1% gelatin (Sigma, Saint Louis, MI, USA) in PBS, cells were immuno-labelled with primary antibodies for 1 h at room temperature. After rinsing in PBS, cells were incubated for 45 min with the appropriate Alexa Fluor-conjugated secondary antibodies. Images were acquired using an epifluorescence microscope (Leika DM6000, Leica-Microsystems, Wetzlar, Germany; https://www.leica-microsystems.com) and a confocal laser-scanning fluorescence microscope (Leica TSC-SP2, Leica-Microsystems, Wetzlar, Germany). The auto contrast function was applied to the captured images as a whole by using Adobe Photoshop CS6 software for a more accurate tonal and colour correction workflow. 

### 4.11. Statistical Analysis

For the quantitative analysis, three independent experiments were performed on different astrocyte primary culture preparations. Statistically significant differences between treated and untreated groups were computed using the analysis of variance for multiple statistical comparison. The statistical software GraphPad Prism version 6.1 was used for statistical analysis. The normal distribution was determined using the D’ata points were excluded. A One-way ANOVA analysis was used with a subsequent Newman–Keuls Multiple Comparison Test for multiple comparisons. Statistical results were shown as scatterplots with the mean ± SEM by using GraphPad Prism 6.1 software. All *p*-values < 0.05 were considered statistically significant. 

## Figures and Tables

**Table 1:** Experimental conditions utilised for synthesising the used PALM.

PALM	Gas	Flow Rate(slm)	V (kV)	Distance (mm)	Period (DC%)	Frequency (kHz)	Treatment Time (s)	Dissipated Energy (J)
P.O_2_	O_2_	0.5	13	3	100 (25)	6	30	51 ± 2
P.Air	Air	0.5	13	3	100 (25)	6	30	46 ± 5
P.N_2_	N_2_	0.5	13	3	100 (25)	6	30	43 ± 0.9

## Supplementary Materials

Supplementary materials can be found at https://www.mdpi.com/1422-0067/21/9/3343/s1.

## References

[1] Allen N.J., Barres B.A. (2009). Neuroscience: Glia—more than just brain glue. Nature.

[2] Sofroniew M.V., Vinters H.V. (2010). Astrocytes: Biology and pathology. Acta Neuropathol..

[3] Iadecola C. (2017). The neurovascular unit coming of age: A journey through neurovascular coupling in health and disease. Neuron.

[4] Sofroniew M.V. (2009). Molecular dissection of reactive astrogliosis and glial scar formation. Trends Neurosci..

[5] Nicchia G.P., Pisani F., Simone L., Cibelli A., Mola M.G., Dal Monte M., Frigeri A., Bagnoli P., Svelto M. (2016). Glio-vascular modifications caused by aquaporin-4 deletion in the mouse retina. Exp. Eye Res..

[6] Eng L.F., Ghirnikar R.S. (1994). GFAP and astrogliosis. Brain Pathol..

[7] Badaut J., Lasbennes F., Magistretti P.J., Regli L. (2002). Aquaporins in brain: Distribution, physiology, and pathophysiology. J. Cereb. Blood Flow Metab..

[8] Taniguchi M., Yamashita T., Kumura E., Tamatani M., Kobayashi A., Yokawa T., Maruno M., Kato A., Ohnishi T., Kohmura E. (2000). Induction of aquaporin-4 water channel mRNA after focal cerebral ischemia in rat. Mol. Brain Res..

[9] Collignon F., Wetjen N.M., Cohen-Gadol A.A., Cascino G.D., Parisi J., Meyer F.B., Marsh W.R., Roche P., Weigand S.D. (2006). Altered expression of connexin subtypes in mesial temporal lobe epilepsy in humans. J. Neurosurg..

[10] Sheng W.S., Hu S., Feng A., Rock R.B. (2013). Reactive oxygen species from human astrocytes induced functional impairment and oxidative damage. Neurochem. Res..

[11] Schreiner B., Romanelli E., Liberski P., Ingold-Heppner B., Sobottka-Brillout B., Hartwig T., Chandrasekar V., Johannssen H., Zeilhofer H.U., Aguzzi A. (2015). Astrocyte depletion impairs redox homeostasis and triggers neuronal loss in the adult CNS. Cell. Rep..

[12] Ben Haim L., Carrillo-de Sauvage M.-A., Ceyzériat K., Escartin C. (2015). Elusive roles for reactive astrocytes in neurodegenerative diseases. Front. Cell. Neurosci..

[13] Finsterwald C., Magistretti P.J., Lengacher S. (2015). Astrocytes: New targets for the treatment of neurodegenerative diseases. Curr. Pharm. Des..

[14] Skaper S.D., Facci L., Zusso M., Giusti P. (2018). An inflammation-centric view of neurological disease: Beyond the neuron. Front. Cell. Neurosci..

[15] Sosunov A., Olabarria M., Goldman J.E. (2018). Alexander disease: An astrocytopathy that produces a leukodystrophy. Brain Pathol..

[16] Maezawa I., Swanberg S., Harvey D., LaSalle J.M., Jin L.-W. (2009). Rett syndrome astrocytes are abnormal and spread MeCP2 deficiency through gap junctions. J. Neurosci..

[17] Meunier C., Merienne N., Jollé C., Déglon N., Pellerin L. (2016). Astrocytes are key but indirect contributors to the development of the symptomatology and pathophysiology of Huntington’s disease. Glia.

[18] Barbeito L. (2018). Astrocyte-based cell therapy: New hope for amyotrophic lateral sclerosis patients?. Stem Cell Res. Ther..

[19] Graves D.B. (2017). Low temperature plasma biomedicine : A tutorial review. Phys. Plasm..

[20] Von Woedtke T., Schmidt A., Bekeschus S., Wende K., Weltmann K.D. (2019). Plasma medicine: A field of applied redox biology. In Vivo.

[21] Wende K., Vandana K.W. (2018). A comparison of floating-electrode DBD and kINPen jet : Plasma parameters to achieve similar growth reduction in colon cancer cells under standardized conditions. Plasma Chem. Plasma Process..

[22] Von Woedtke T., Metelmann H.R., Weltmann K.D. (2014). Clinical plasma medicine: State and perspectives of in vivo application of cold atmospheric plasma. Contrib. Plasma Phys..

[23] Reuter S., Von Woedtke T., Weltmann K.D. (2018). The kINPen—A review on physics and chemistry of the atmospheric pressure plasma jet and its applications. J. Phys. D Appl. Phys..

[24] 24 Trizio I., Trulli M.G., Lo Porto C., Pignatelli D., Camporeale G., Palumbo F., Sardella E., Gristina R., Favia P. (2018). Plasma processes for life sciences. Chem. Mol. Sci. Chem. Eng..

[25] Fiebrandt M., Lackmann J.W., Stapelmann K. (2018). From patent to product: 50 years of low-pressure plasma sterilization. Plasma Processes Polym..

[26] Anderson C.E., Cha N.R., Lindsay A.D., Clark D.S., Graves D.B. (2016). The role of interfacial reactions in determining plasma-liquid chemistry. Plasma Chem. Plasma Process..

[27] Bruggeman P.J., Kushner M.J., Locke B.R., Gardeniers J.G.E., Graham W.G., Graves D.B., Hofman-Caris R.C.H.M., Maric D., Reid J.P., Ceriani E. (2016). Plasma-liquid interactions: A review and roadmap. Plasma Sources Sci. Technol..

[28] Kaushik N.K., Ghimire B., Li Y., Adhikari M., Veerana M., Kaushik N., Jha N., Adhikari B., Lee S.J., Masur K. (2018). Biological and medical applications of plasma-activated media, water and solutions. Biol. Chem..

[29] Von Woedtke T., Haertel B., Weltmann K.D., Lindequist U. (2013). Plasma pharmacy—Physical plasma in pharmaceutical applications. Pharmazie.

[30] Katiyar K.S., Lin A., Fridman A., Keating C.E., Cullen D.K., Miller V. (2019). Non-thermal plasma accelerates astrocyte regrowth and neurite regeneration following physical trauma in vitro. Appl. Sci..

[31] Bauer G., Sersenová D., Graves D.B., Machala Z. (2019). Cold atmospheric plasma and plasma-activated medium trigger RONS-based tumor cell apoptosis. Sci. Rep..

[32] Haskew-Layton R.E., Payappilly J.B., Smirnova N.A., Ma T.C., Chan K.K., Murphy T.H., Guo H., Langley B., Sultana R., Butterfield D.A. (2010). Controlled Enzymatic Production of Astrocytic Hydrogen Peroxide Protects Neurons From Oxidative Stress via an Nrf2-independent Pathway. Proc. Natl. Acad. Sci. USA.

[33] Sakiyama Y., Graves D.B., Chang H., Shimizu T., Morfill G. (2012). Plasma chemistry model of surface microdischarge in humid air and dynamics of reactive neutral species. J. Phys. D Appl. Phys..

[34] Tarabová B., Lukeš P., Hammer M.U., Jablonowski H., von Woedtke T., Reuter S., Machala Z. (2019). Fluorescence measurements of peroxynitrite/peroxynitrous acid in cold air plasma treated aqueous solutions. Phys. Chem. Chem. Phys..

[35] Te Boekhorst V., Preziosi L., Friedl P. (2016). Plasticity of cell migration in vivo and in silico. Annu. Rev. Cell. Dev. Biol..

[36] Hsieh H.L., Wang H.H., Wu W.B., Chu P.J., Yang C.M. (2010). Transforming growth factor-β1 induces matrix metalloproteinase-9 and cell migration in astrocytes: Roles of ROS-dependent ERK- and JNK-NF-κB pathways. J. Neuroinflamm..

[37] Azzariti A., Iacobazzi R.M., Di Fonte R., Porcelli L., Gristina R., Favia P., Fracassi F., Trizio I., Silvestris N., Guida G. (2019). Plasma activated medium as anticancer tool and immunogenic cell death inducer in metastatic melanoma and pancreatic cancer models. Sci. Rep..

[38] Van Boxem W., Van der Paal J., Gorbanev Y., Vanuytsel S., Smits E., Dewilde S., Bogaerts A. (2017). Anti-cancer capacity of plasma-treated PBS: Effect of chemical composition on cancer cell cytotoxicity. Sci. Rep..

[39] Xiong Z., Zhao S., Mao X., Lu X., He G., Yang G., Chen M., Ishaq M., Ostrikov K. (2014). Selective neuronal differentiation of neural stem cells induced by nanosecond microplasma agitation. Stem Cell Res..

[40] Omar S.A., Artime E., Webb A.J. (2012). A comparison of organic and inorganic nitrates/nitrites. Nitric Oxide.

[41] Graves D.B. (2014). Reactive species from cold atmospheric plasma: Implications for cancer therapy. Plasma Processes Polym..

[42] Picón-Pagès P., Garcia-Buendia J., Muñoz F.J. (2019). Functions and dysfunctions of nitric oxide in brain. Biochim. Biophys. Acta Mol. Basis Dis..

[43] Goswami P., Gupta S., Joshi N., Sharma S., Singh S. (2015). Astrocyte activation and neurotoxicity: A study in different rat brain regions and in rat C6 astroglial cells. Environ. Toxicol. Pharmacol..

[44] Judée F., Fongia C., Ducommun B., Yousfi M. (2016). Short and long time effects of low temperature plasma activated media on 3D multicellular tumor spheroids. Sci. Rep..

[45] Mola M.G., Sparaneo A., Gargano C.D., Spray D.C., Svelto M., Frigeri A., Scemes E., Nicchia G.P. (2016). The speed of swelling kinetics modulates cell volume regulation and calcium signaling in astrocytes: A different point of view on the role of aquaporins. Glia.

[46] De Bellis M., Pisani F., Mola M.G., Basco D., Catalano F., Nicchia G.P., Svelto M., Frigeri A. (2014). A novel human aquaporin-4 splice variant exhibits a dominant-negative activity: A new mechanism to regulate water permeability. Mol. Biol. Cell.

