# Supplementary figures and images for: A Synergistic Effect of Reactive Oxygen and Reactive Nitrogen Species in Plasma Activated Liquid Media Triggers Astrocyte Wound Healing
Source: Int J Mol Sci. 2020 May 8;21(9):3343. doi: 10.3390/ijms21093343 (PMC7247562; doi:10.3390/ijms21093343)

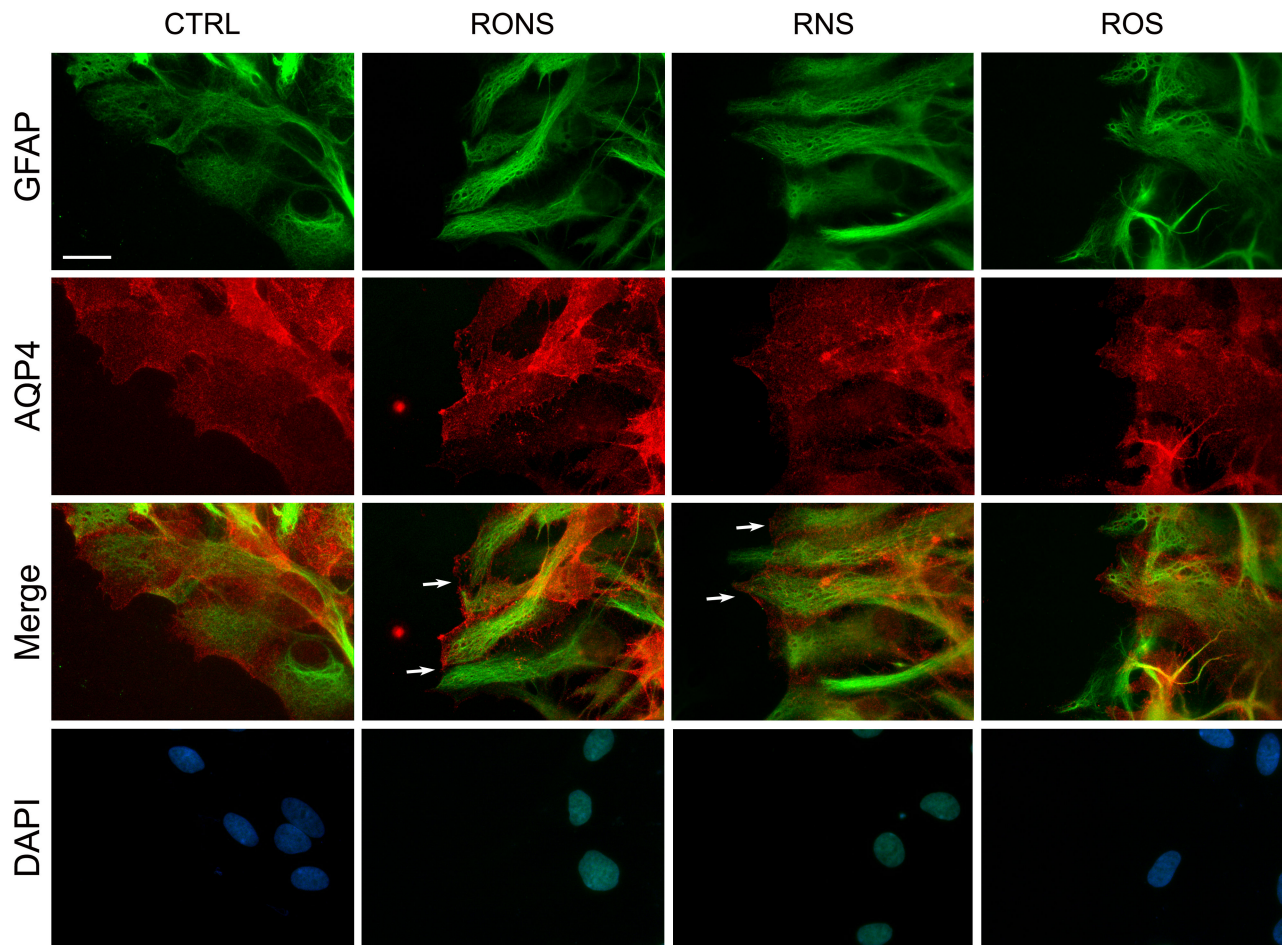

Supplement: Supplementary file 1 [file ijms-21-03343-s001.pdf]
